# DNA methyltransferase 1 knockdown reverses PTEN and VDR by mediating demethylation of promoter and protects against renal injuries in hepatitis B virus-associated glomerulonephritis

**DOI:** 10.1186/s13578-022-00835-1

**Published:** 2022-06-28

**Authors:** Haochen Guan, Nan Zhu, Gang Tang, Yi Du, Ling Wang, Weijie Yuan

**Affiliations:** Department of Nephrology, Shanghai General Hospital, Shanghai Jiao Tong University School of Medicine, 100 Haining Road, 200080 Shanghai, People’s Republic of China

**Keywords:** Hepatitis B virus-associated glomerulonephritis, Hepatitis B virus X protein, DNA methylation, Phosphatase and tensin homolog, Vitamin D receptor

## Abstract

**Background:**

Aberrant DNA methylation patterns, including hypermethylation of key genes that inhibit fibrosis and inflammation, have been described in human kidney diseases. However, the role of DNA methyltransferase 1 (DNMT1) in hepatitis B virus-associated glomerulonephritis (HBV-GN) remains unclear.

**Methods:**

We explored the underlying mechanism by establishing HBV X protein (HBx) overexpressing renal tubular epithelial (HK-2) cells and human podocytes with DNMT1 knockdown. Using RNA-sequencing to determine the downstream targets of DNMT1 and evaluate its levels of promoter methylation. HBV transgenic mice were used to examine the effects of DNMT1 inhibitor on renal in vivo.

**Results:**

DNMT1 was significantly upregulated in the renal tissue of HBV-GN patients, accompanied by injuries of HK-2 cells and podocytes. HBx markedly upregulated DNMT1 and induced epithelial-mesenchymal transition (EMT) and inflammation in HK-2 cells and human podocytes. This increased DNMT1 expression was attenuated after DNMT1 knockdown, accompanied by restored HK-2 cells and podocyte injuries resulting from the activation of PI3K/Akt/mTOR and nuclear factor-kappa B (NF-κB) pathways. Hypermethylation of the phosphatase and tensin homolog (PTEN) promoter and vitamin D receptor (VDR) was induced in HBx-overexpressing HK-2 cells and podocytes, respectively, whereas DNMT1 knockdown effectively corrected these alterations. Furthermore, PTEN and VDR ablation resulted in marked EMT and inflammation induction in HBx-overexpressing HK-2 cells and human podocytes even with DNMT1 knockdown. Downregulation of the PI3K/Akt/mTOR-related pathway attenuated HBx-induced EMT and inflammation in HK-2 cells. Luciferase reporter assay revealed VDR as a direct target of the Snail family transcriptional repressor 1 (SNAI1) in HBx-overexpressing podocytes. DNA methylation inhibitor 5-azacytidine alleviated urinary protein and renal inflammation in HBV transgenic mice via PTEN-PI3K/Akt signaling and VDR signaling axis.

**Conclusions:**

Our study clarifies the potential epigenetic mechanisms underlying HBx-induced renal injuries in HBV-GN and the renoprotective effects of inhibiting DNMT1, which can provide important insights into the development of treatments for HBV-GN.

**Supplementary Information:**

The online version contains supplementary material available at 10.1186/s13578-022-00835-1.

## Introduction

Hepatitis B virus (HBV) infection is widespread worldwide and manifests as several extrahepatic syndromes [1]. HBV-associated glomerulonephritis (HBV-GN) is a cause of HBV-induced renal impairment and is one of leading secondary renal diseases in China. HBV-GN usually manifests as glomerular damage; its main pathological types are membranous, membranous proliferative, IgA, and mesangial proliferative nephropathies [2]. Podocytes that reside on glomerular capillaries and play an essential role in the glomerular filtration barrier have been identified as the main targets in the pathogenesis of HBV-GN. In contrast, accumulating evidence suggests that renal tubular epithelial cell (RTEC) injury, tubular atrophy, inflammatory cell infiltration, and tubular interstitial fibrosis in the renal tissue of HBV-GN patients play a critical role function in developing HBV-GN [3, 4]. Therefore, determining the causative mechanisms of HBV-GN, especially targeting podocytes and renal tubular injury, has become an urgent problem in the clinic.

HBV-GN is generally believed to be caused by the deposition of immune complexes. However, recent studies have revealed that direct virus-induced renal pathological alterations contribute to the HBV-GN progression in addition to immune dysfunction. The HBV X protein (HBx), a small soluble protein comprising 154 amino acids, plays the most important regulatory role in HBV transcription and replication [5]. HBx is a multifunctional transactivator that affects cell transcription, signal transduction, cell cycle regulation, DNA repair, apoptosis, and chromosomal stability [6, 7]. It modulates AP-1 and nuclear factor-kappa B (NF-κB) transcription and activates the JAK/STAT, Ras/Raf/MAPK, JNK, and PI3K/Akt signaling pathways to promote disease progression [8]. HBx has been identified within the glomeruli and tubular epithelial cells of HBV-GN patients, suggesting a role for HBx in podocyte and tubulointerstitial damage. Previous studies have revealed that HBx enhances tumor necrosis factor‒related apoptosis-inducing ligand (TRAIL)-induced apoptosis of RTECs by enhancing NF-κB activation, resulting in the enhanced production of proinflammatory cytokines such as TNF-α and IFN-γ [9]. HBx also stimulates renal epithelial-mesenchymal transition (EMT) via the activation of NF-κB [10]. However, the precise pathogenic mechanism underlying HBx-induced renal injuries has not been fully clarified.

Epigenetic modifications, i.e., genomic DNA methylation and histone modifications, are associated with various renal disease etiologies [11,12]. Recent research has found that histone demethylase promotes renal inflammation by mediating TLR4 signaling in HBV-GN, thereby revealing epigenetic modifications associated with HBV-induced renal injuries [13]. In DNA methylation—an indispensable modification associated with epigenetic regulation—DNA methyltransferases (DNMTs) deposit methyl groups at the CpG dinucleotides. DNMT1 maintains the DNA methylation levels during DNA replication and repair and is responsible for transmitting the DNA methylation information to progeny cells, as it is a pivotal factor in maintaining promoter methylation. Further, it is known to play a role in the differentiation and development of inflammatory cells. Moreover, blocking of DNMT1 activity has been reported to inhibit macrophage polarization [14]. In addition, DNA methylation by DNMT1 could inhibit TRIM22 expression, thus causing HBV to evade the IFN- and TRIM22-associated antiviral machinery [15]. Therefore, DNMT1 is believed to be involved in cell proliferation, differentiation, apoptosis, and viral escape and is closely associated with tumor metastasis, inflammation, and viral replication. Several studies indicated that hypermethylation of various promoters was related to the pathogenesis of acute and chronic kidney diseases, and that the reversibility of epigenetic modifications offered a molecular foundation for epigenetic intervention in the treatment of kidney diseases [16–18]. However, the exact mechanism by which DNMT1 regulates HBx-induced podocyte and RETC injury remains unclear.

This study aimed to assess the potential mechanism of action DNMT1 in HBx-induced renal inflammation and fibrosis in HBV-GN. We found that DNMT1 promoted HBx-induced fibrosis and proinflammatory mediator production by enhancing the promoter methylation of phosphatase and tensin homolog (PTEN) and vitamin D receptor (VDR) in RTECs and human podocytes, respectively. Furthermore, DNMT1 knockdown effectively recovered PTEN and VDR promoter hypermethylation, thereby contributing to the downregulation of PI3K/Akt/mTOR and NF-κB signaling pathways, which could restore the protein loss and protect against HBx-induced renal injuries in vivo and in vitro. Therefore, our study explored epigenetic mechanisms—from the perspective of fibrosis and inflammation in HBx-induced renal dysfunction—leading to the development of HBV-GN.

## Materials and methods

### Patient samples

Kidney biopsy samples were obtained from 15 inpatients diagnosed with HBV-GN and 50 inpatients without HBV-GN. HBV-GN was diagnosed based on the following criteria: (a) serum hepatitis B surface antigen (HBsAg) positivity, (b) glomerular nephritis, where secondary glomerular disorders and lupus nephritis were excluded, and (c) HBV DNA or HBV antigen positivity in renal samples. Patients were classified into four groups (groups I–IV), i.e., HBV-GN (Group I, n = 15), HBV-positive PGN (Group II, n = 20, PGN cases showing positive serum HBV DNA or HBsAg results), HBV-negative PGN (Group III, n = 15, PGN cases not affected by HBV), and normal control (n = 15, matched non-carcinoma kidney tissue samples). This study was conducted in accordance with the Second Declaration of Helsinki. Each patient provided informed consent.

### Animals

Fourteen 6-week-old HBV-Tg C57BL/6 male mice (HBV mice) and age-matched WT C57BL/6 male mice (C57 mice) were obtained from Vitalstar Biotechnology Co., Ltd. (Beijing, China). Each mouse was raised in an SPF environment under a 12-hour/12-hour light/dark cycle at 22 °C. Animals were allowed ad libitum access to drinking water. Half of the HBV (n = 7) and C57 mice (n = 7) were randomly chosen to receive 5-azacytidine dissolved in PBS (5-Aza, Sigma-Aldrich, St. Louis, MO, USA) at 16 weeks of age. These mice were intraperitoneally injected with 5-Aza (1 mg/kg body weight) three times per week. The same volume of PBS was intraperitoneally injected into the control HBV (n = 7) and C57 mice (n = 7). After 8 weeks of treatment, 24 h urine, blood, and kidney samples were collected. All experiments involving animals were approved by the Animal Care and Use Committee of Shanghai General Hospital, Shanghai Jiaotong University School of Medicine. And we declared that each experiment was carried out according to the guidelines for the Care and Use of Laboratory Animals (NIH publication, Eighth edition, 2011) released by the National Institutes of Health.

### Assessment of renal function

The total blood samples were centrifuged at 3500 × g for 15–30 min. Serum creatinine (Scr) levels were determined with an automatic biochemical analyzer. The 24 h urine samples were collected from metabolic cages, and urinary protein (Upro) was determined with an automatic biochemical analyzer.

### Cell culture

Human tubular epithelial cells, i.e., HK-2 cells were obtained from the American Type Culture Collection (ATCC), and cultured in DMEM/F12 (Gibco, NY, USA) supplemented with 1% penicillin/streptomycin (Sigma-Aldrich, St. Louis, MO, USA) and 10% fetal bovine serum (FBS, Gibco, NY, USA) and incubated in a humid atmosphere of 5% CO_2_ at 37 °C. The conditionally immortalized human podocyte cell line demonstrating nephrin and podocin expression was kindly provided by Prof. Chuanming Hao (Huashan Hospital, Fudan University) and cultured as previously described [19]. Differentiated podocytes were cultured in RPMI-1640 medium (Gibco, NY, USA), containing 1% penicillin/streptomycin and 10% FBS.

### Plasmid and RNA interference

Full-length sequences of the gene encoding the HBx (Gene ID: 944,566) were chemically synthesized, and these were then subcloned into a lentiviral expression vector (PGMLV-PA6) obtained from Genomeditech (Shanghai, China). The sequences of the DNMT1, VDR, and PTEN genes were obtained from GenBank. Genepharma Co., Ltd (Genepharma, Shanghai, China) designed and synthesized control siRNA and siRNA against DNMT1, VDR, PTEN. siSNAI1 was purchased from Santa Cruz (sc-38398, CA, USA) (Additional file 1: Table S1).

### Transfection and stable cell line generation

Cells were seeded in 6-well plates (1 × 10^5^/well), and when the cell density reached approximately 70–80%, siRNA or plasmids were transfected into cells using Lipofectamine 3000 (Invitrogen, Carlsbad, CA, USA) in accordance with the manufacturer’s instructions. HBx overexpression and control lentiviral vectors—containing a random sequences—were transfected into HEK 293 T cells. After 48 h of transfection, the medium containing the lentivirus was harvested and used for infecting the HK-2 cells and human podocytes, followed by a week of puromycin (2 µg/mL) selection to generate stably transfected cells. Cells with stable HBx expression were considered HBx-HK-2 cells or HBx-podocytes, whereas those with stable empty vector were used as negative control (NC) cells. The untreated cells were called control (Cont) cells.

### RNA-sequencing and transcriptome analysis

NC cells transfected with siNC were used as negative controls. HBx-HK-2 cells or HBx-podocytes transfected with or without siDNMT1 were used to analyze the potential downstream targets of DNMT1. TRIzol (Invitrogen, Carlsbad, CA, USA) was used to extract total RNA, and libraries were established using the TruSeq Stranded mRNA LTSample Prep Kit (Illumina, San Diego, CA, USA) in accordance with the manufacturer’s instructions. The constructed libraries were then sequenced using the Illumina sequencing platform (Illumina HiSeq X Ten or HiSeqTM 2500), which produced paired-end reads of 125 bp/150 bp. Transcriptomic analysis and sequencing was outsourced to OE Biotech Co., Ltd. (Shanghai, China). *P* < 0.05, and fold change (FC) < 0.5 or FC > 2 indicated significant differential expression.

### Immunofluorescence

After rinsing twice with PBS, cells were fixed for 30 min in 4% paraformaldehyde, permeabilized using 0.2% Triton X-100, and blocked using 0.5% bovine serum albumin (BSA) at room temperature. Thereafter, cells were probed overnight with anti-DNMT1 (#ab188453), anti-VDR (#ab89626), anti-PTEN (#ab137337) (Abcam, Cambridge, MA, UK), anti-DNMT1 (#sc-271,729), anti-SNAI1 (#sc-271,977), anti-nephrin (#sc-376,522) (Santa Cruz, CA, USA), anti-E-cadherin (#14,472), and anti-p-NF-κB P65 (#3033) (Cell Signaling Technology, Danvers, MA, USA), anti-HBx (#MA1-81021) (Thermo Fisher Scientific, Waltham, MA, USA) antibodies at 4 °C. This was followed by a 60 min incubation with fluorescence-conjugated secondary antibodies (Yeasen, Shanghai, China) in the dark at room temperature. Then, cells were stained with DAPI (Beyotime, Shanghai, China) for 10 min at room temperature.

### Immunohistochemistry

Human and animal kidney samples were fixed with 4% paraformaldehyde, embedded in paraffin, and sliced into 4 μm sections. After blocking with 5% BSA at room temperature, the sections were probed overnight with antibodies against HBx (#ab235), IL-6 (#ab9324, #ab208113), DNMT1 (#ab188453) (Abcam, Cambridge, MA, UK), and TNF-α (#sc-52,746) (Santa Cruz, CA, USA) at 4 °C, followed by incubation with appropriate secondary antibodies. The sections were visualized using a light microscope (Leica, Heidelberg, Germany) and quantified using ImageJ.

### Masson’s trichromatic staining

Kidney sections were prepared as described above and then dewaxed and hydrated. After rinsing with water, the sections were stained using reagents from a Masson Modified International Medical Equipment (IMEB) Stain Kit (IMEB, San Marcos, CA, USA), in accordance with the manufacturer’s instructions. Collagen deposition was imaged using a microscope (Leica, Heidelberg, Germany).

### Quantitative real-time PCR

TRIzol was used to isolate the total RNA. Thereafter, 1 µg of total RNA was reverse transcribed into cDNA using the Toyobo reverse transcription system (Osaka, Japan). SYBR Green Premix (Takara, Otsu, Japan) was used to conduct quantitative real-time PCR **(**qRT-PCR) on an ABI 7700 Sequence Detector System (Applied Biosystems, USA). The expression of target genes was normalized to the expression of GAPDH. We used the 2^−ΔΔCT^ method to determine the fold-change at the target gene level. Additional file 1: Table S2 displays all the primers used.

### Protein isolation and western-blotting

RIPA buffer (Thermo Fisher Scientific, Waltham, MA, USA), supplemented with phosphatase/protease inhibitors (Thermo Fisher Scientific, Waltham, MA, USA), was used to lyse cells and tissues. Proteins were collected after centrifuging the lysate at 15,000 × g for 15 min. Equivalent amounts of protein were loaded into each well of 6–12% SDS-PAGE gels, and then transferred onto a PVDF membrane (Thermo Fisher Scientific, Waltham, MA, USA). Thereafter, the membranes were blocked in 5% skim milk for 1–2 h at room temperature, followed by overnight incubation at 4 °C with primary antibodies, i.e., anti-HBx (#ab235), anti-DNMT1 (#ab188453), anti-DNMT3a (#ab188470), anti-DNMT3b (#ab2851), anti-ZO1 (#ab216880), anti-VDR (#ab3508), anti-PTEN (#ab137337), anti-podocin (#ab181143), anti-fibronectin (#ab2413), anti-IL-6 (#ab9324, #ab208113)(Abcam, Cambridge, MA, UK), anti-p-NF-κB P65 (#3033), anti-NF-κB P65 (#8242), anti-p-IκBα (#2859), anti-IκBα (#4814), anti-E-cadherin (#14,472), anti-vimentin (#5741), anti-p-mTOR (#5536), anti-mTOR (#2983), anti-p-Akt (#5012), anti-Akt (#4691), anti-β-actin (#3700), and anti-GAPDH (#5174) (Cell Signaling Technology, Danvers, MA, USA), and anti-SNAI1 (#sc-271,977), anti-nephrin (#sc-376,522) and anti-TNF-α (#sc-52,746) (Santa Cruz, CA, USA). This was followed by a 2 h incubation with horseradish peroxidase-conjugated secondary anti-rabbit IgG (#7074) and anti-mouse IgG (#7076) (Cell Signaling Technology, Danvers, MA, USA) under room temperatures. ECL (Millipore, Billerica, MA, USA) was used to visualize and analyze the protein bands.

### Enzyme-linked immunosorbent assay

Commercial enzyme-linked immunosorbent assay (ELISA) kits (R&D, Minneapolis, MN, USA) were used to measure TNF-α and IL-6 levels in the supernatant, in accordance with the manufacturers’ instructions. TNF-α and IL-6 levels were analyzed based on OD_450_.

### Bisulfite-sequencing PCR

Total genomic DNA was isolated from HK-2 cells and human podocytes. After bisulfite treatment, 1 µg genomic DNA was modified using the EpiTect Bisulfite Kit (Qiagen, Germantown, MD, USA), followed by PCR amplification using Bisulfite-sequencing PCR (BSP) forward and reverse primers (Additional file 1: Table S3). After PCR amplification procedure, the PCR products corresponding to the CpG islands of the PTEN and VDR promoters (351 base pairs and 399 base pairs) were subjected to gel purification and insertion into the pMD18-T vector. Ten clones were chosen for sequencing after transformation into TOP10 (DL1010) competent cells.

### Dual-luciferase reporter assay

Cells were seeded into 24-well plates, followed by co-transfection with pcDNA3.1/Flag-SNAI1, pGL4.10-VDR luciferase promoter-reporter plasmid (wild-type or mutant plasmid), and a plasmid encoding Renilla luciferase (pRL-TK plasmid, OBiO Technology, Shanghai, China). After 48 h, cells were harvested, and Renilla and firefly luciferase activities were assessed—using the Dual-Luciferase Reporter Assay System purchased from Promega (Madison, WI, USA)—based on a previous description [20]. The firefly luciferase to Renilla luciferase ratio was calculated.

### Nuclear extract preparation

Nuclear extracts were prepared using a nuclear protein extraction kit (Beyotime, Shanghai, China) in accordance with the manufacturer’s instruction. Briefly, cells were rinsed twice with PBS and resuspended in reagent A to extract the cytoplasmic protein fraction. After extraction of cytoplasmic proteins, the pellets were resuspended in 50 µL nuclear protein extraction reagent. After agitation at 4 °C and centrifugation at 12,000×*g* for 10 min, the supernatant containing the nuclear proteins was harvested. Nuclear extracts were used for further analysis or stored at − 80 °C.

### DNMT1 activity assay

Ten to twenty micrograms of the nuclear extract was harvested, and DNMT1 activity was evaluated using the DNMT1 assay kit (Abcam, Cambridge, MA, UK) in accordance with the manufacturer’s protocol. DNMT activity based on OD_450_ values was measured using a microplate reader.

### Statistical analysis

Each experiment was performed with three or more independent replicates. All data are shown as the mean ± SD and were compared using one-way ANOVA or Student’s *t*-test. GraphPad Prism 7.0 (GraphPad Software, Inc., Jolla, CA, USA) was used for graphical visualization and statistical analyses. Significance was set at *P* < 0.05.

## Results

### Significantly upregulated DNMT1 levels in HBx-overexpressing renal cells and renal tissues of HBV-GN patients

**Fig. 1:**
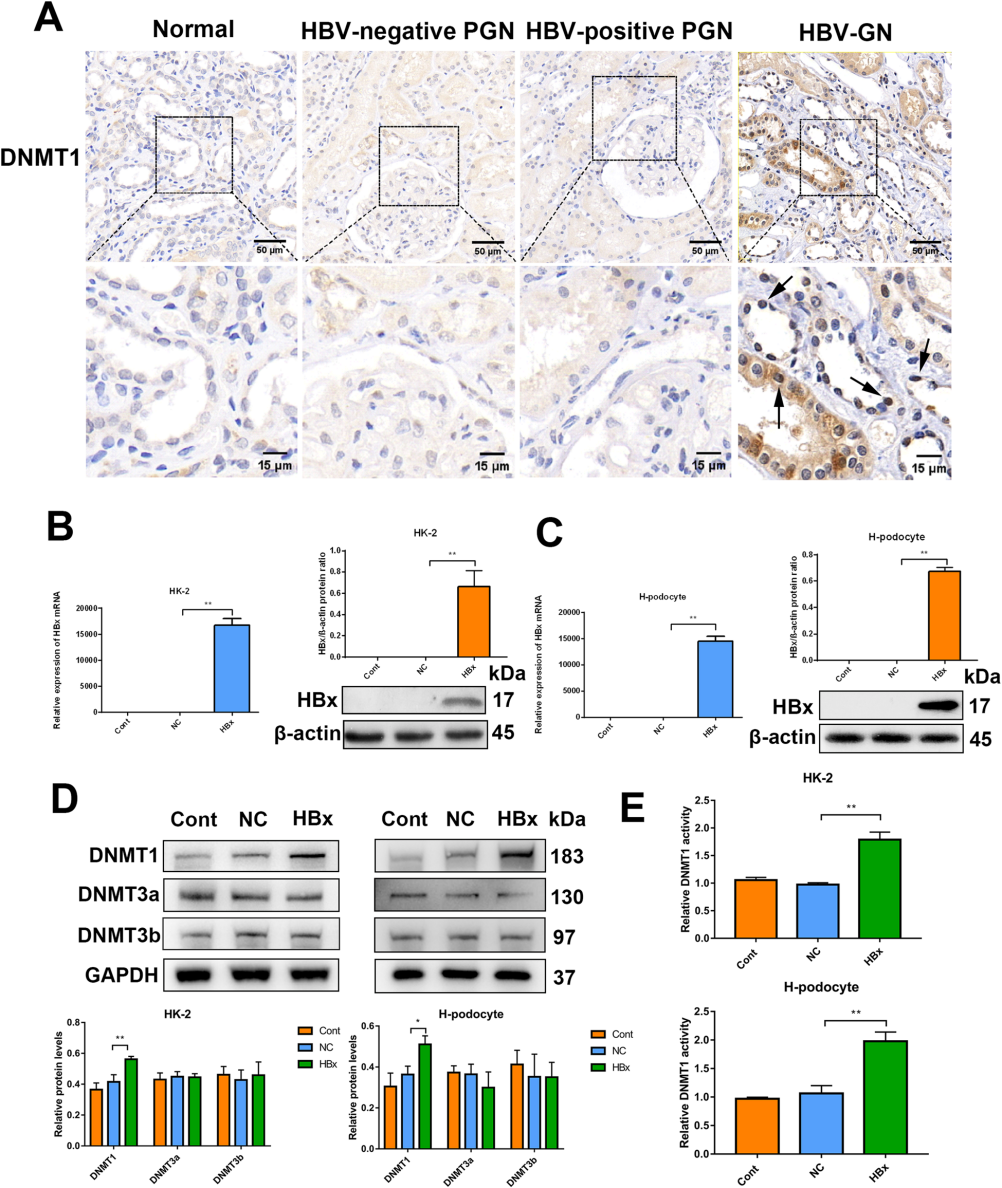
DNMT1 upregulation among HBV-GN patients and HBx-transfected renal cells. **A** DNMT1 expression was determined using immunohistochemistry. **B** and **C** HK-2 cells and human podocytes transfected with empty vector lentivirus (NC) or HBx-expressing lentivirus (HBx) and without transfection (Cont) were examined for HBx expression by western blotting and RT-qPCR. **D** The expression of DNMT1, DNMT3a, and DNMT3b was measured in HK-2 cells and human podocytes using western blotting. **E** DNMT1 activity was evaluated using the DNMT1 assay kit. Data are expressed as mean ± SD of 3 separate assays. *P < 0.05; **P < 0.01

To investigate the function of DNMT1 in HBV-GN, we collected renal biopsy samples from 15 HBV-GN patients, 15 HBV-antigen-negative PGN patients, 20 HBV-antigen-positive PGN patients, and 15 matched non-carcinoma samples. In the normal control group, the DNMT1 expression was so low that it could not be detected by immunohistochemistry (IHC) (Fig. 1 A). In the HBV-antigen-negative and HBV-antigen-positive PGN groups, a slight increase in DNMT1 expression was detected (Fig. 1 A). DNMT1 showed markedly increased expression in the HBV-GN group than in the non-HBV-GN group and was found to be localized in the cytoplasm and nuclei of renal tubular epithelial cells (Fig. 1 A). HBx is a multifunctional viral protein that plays a critical role in disease pathogenesis. To analyze whether HBx affects DNMT1 expression in HBV-associated glomerulonephritis, we generated stable HBx-overexpressing cells, HBx-HK-2 cell lines, and HBx-podocytes. We determined the overexpression efficiency of the lentiviral vectors with respect to HBx using qRT-PCR and western blotting (Fig. 1B and C). We then confirmed the expression of three major DNMTs (DNMT1, DNMT3a, and DNMT3b) with or without HBx overexpression in HK-2 cells and human podocytes by western blotting. HBx markedly increased DNMT1 levels (compared to those in control lentiviral vector-transfected cells (NC) or wild-type cells (Cont)) (Fig. 1D). Consistent with these observations, HBx significantly increased the nuclear activity of DNMT1 in HK-2 cells and human podocytes (Fig. 1E). Further, DNMT1 was found to be upregulated in HBx-overexpressing cultured cells and kidney biopsies from HBV-GN patients.

### DNMT1 knockdown protects against injuries in HBx-overexpressing renal cells

**Fig. 2:**
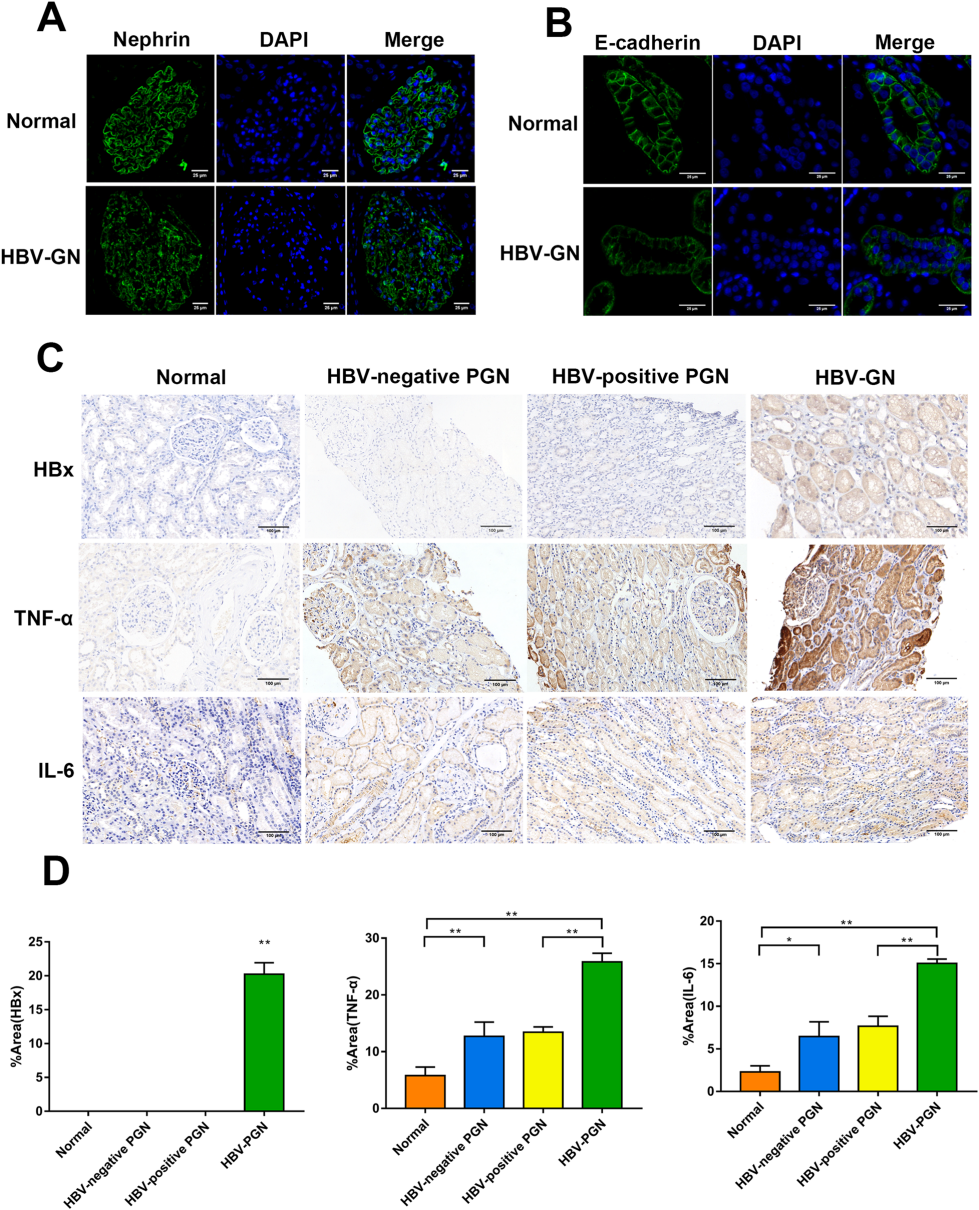
Kidney injuries in HBV-GN patients. **A** Nephrin expression in podocytes analyzed using immunofluorescence. Scale bar: 25 μm. **B** E-cadherin expression in renal tubular epithelial cells was analyzed using immunofluorescence. Scale bar: 25 μm. **C** The expression of HBx, TNF-α, and IL-6 was observed using immunohistochemistry. Scale bar: 100 μm. **D** Analysis of mean staining intensity in each group. Results are expressed as mean ± SD of 3 separate assays. *P < 0.05; **P < 0.01

To further explore the effects of DNMT1 with respect to modulating renal dysfunction in vitro, we first analyzed the expression of the podocyte slit diaphragm protein nephrin, epithelial membrane protein E-cadherin, and proinflammatory mediators in renal biopsies. Compared with the non-HBV GN groups, the HBV-GN group exhibited a significant reduction in glomerular nephrin levels, together with continuous linear pattern loss down the glomerular basement membrane (Fig. 2A). E-cadherin levels in the renal epithelium also decreased significantly in the HBV-GN group (Fig. 2B). IHC revealed the absence of HBx expression in the kidney biopsy samples of non-HBV GN groups, and increased HBx expression in the kidney biopsy samples of HBV-GN groups (Fig. 2C). The expression of proinflammatory factors, including IL-6 and TNF-α, was markedly upregulated in the HBV-GN group (Fig. 2D). Semi-quantitative analysis of the staining intensities confirmed the upregulation of HBx, TNF-α, and IL-6 in the HBV-GN group (compared to the non-HBV-GN group) (Fig. 2D). The above results demonstrate that the expression of DNMT1 in the kidney of HBV-GN patients is significantly higher than that in the HBV-positive PGN group and HBV-negative PGN group, suggesting that the abnormal expression of DNMT1 is closely related to the presence of renal HBV.

**Fig. 3:**
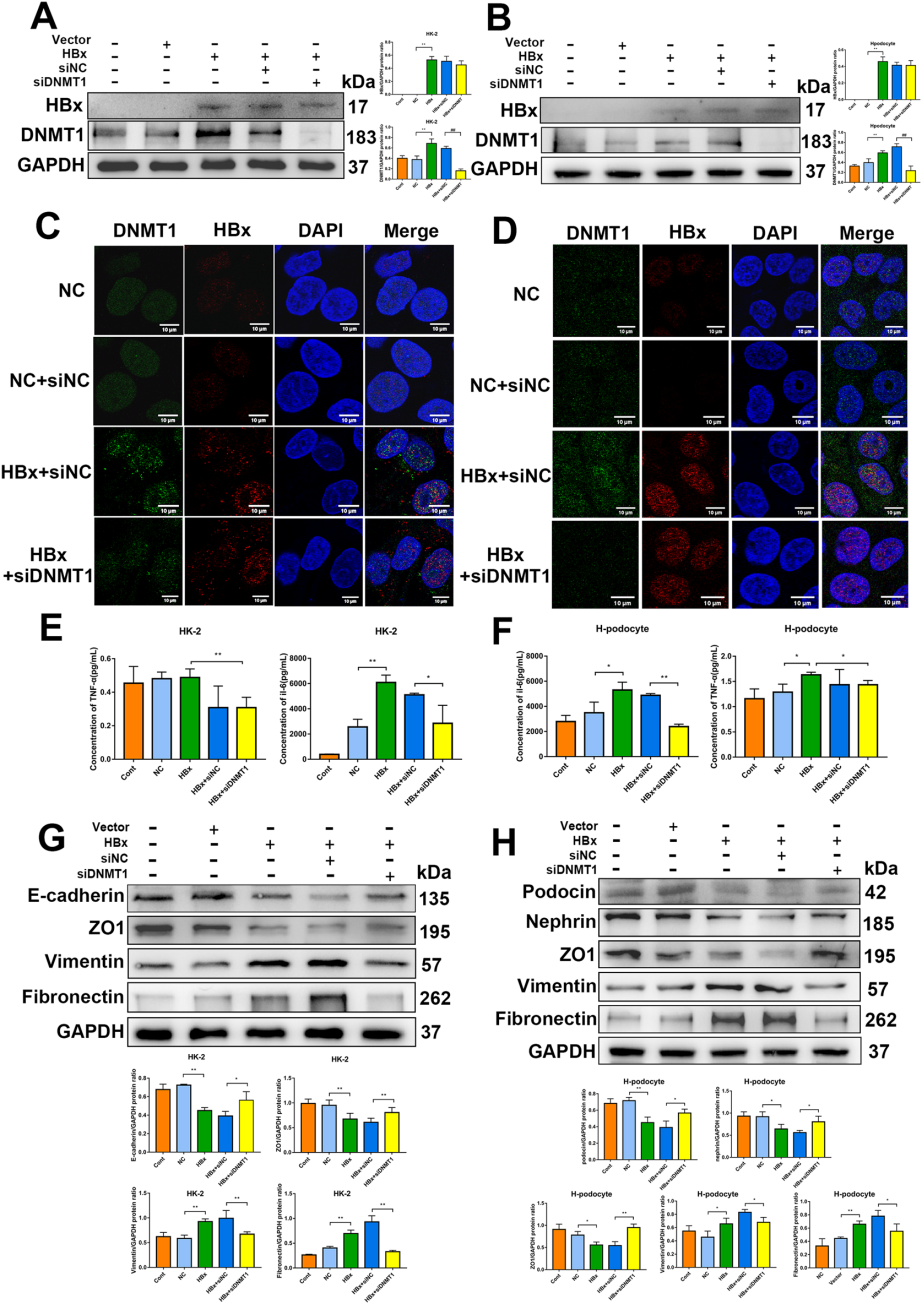
DNMT1 inhibition attenuated HK-2 cell and human podocyte injury in vitro. **A** and **B** The expression of HBx and DNMT1 in HBx-HK-2 cells and HBx-podocytes with or without DNMT1 knockdown was evaluated by western blotting. **C** and **D** The localization and expression of HBx and DNMT1 in HBx-HK-2 cells and HBx-podocytes were determined by immunofluorescence. Scale bar: 10 μm. **E** and **F** ELISA was conducted to measure concentrations of IL-6 and TNF-α in the culture supernatant of HBx-HK-2 cells and HBx-podocytes with or without DNMT1 knockdown. **G** and **H** Western blotting was conducted to measure the expression of E-cadherin, ZO1, Vimentin, Fibronectin, Podocin, and Nephrin in HBx-HK-2 cells and HBx-podocytes with or without DNMT1 knockdown. Results are expressed as mean ± SD of 3 separate assays. *P < 0.05; **P < 0.01

We then tested DNMT1 regulation in the context of kidney injuries. Western blotting revealed that the targeted siRNA effectively knocked down DNMT1 expression in HK-2 cells and human podocytes but did not have any effect on HBx expression (Fig. 3 A and B). Immunofluorescence revealed that HBx and DNMT1 co-localized in the nucleus, and the expression trends corresponded to those observed in western blotting (Fig. 3 C and D). Furthermore, DNMT1 knockdown significantly reversed the HBx-induced release of TNF-α and IL-6 (Fig. 3E and F). Western blotting revealed that HBx downregulated epithelial markers, such as E-cadherin, ZO-1, nephrin, and podocin, whereas it upregulated mesenchymal markers, such as vimentin and fibronectin (Fig. 3G and H). DNMT1 knockdown in the background of HBx overexpression abolished the abnormal expression of EMT markers (Fig. 3G and H). Collectively, these results suggest that DNMT1 promotes HBx-induced kidney injury in vitro.

### RNA-sequencing after DNMT1 knockdown in HBx-overexpressing HK-2 cells and podocytes

**Fig. 4:**
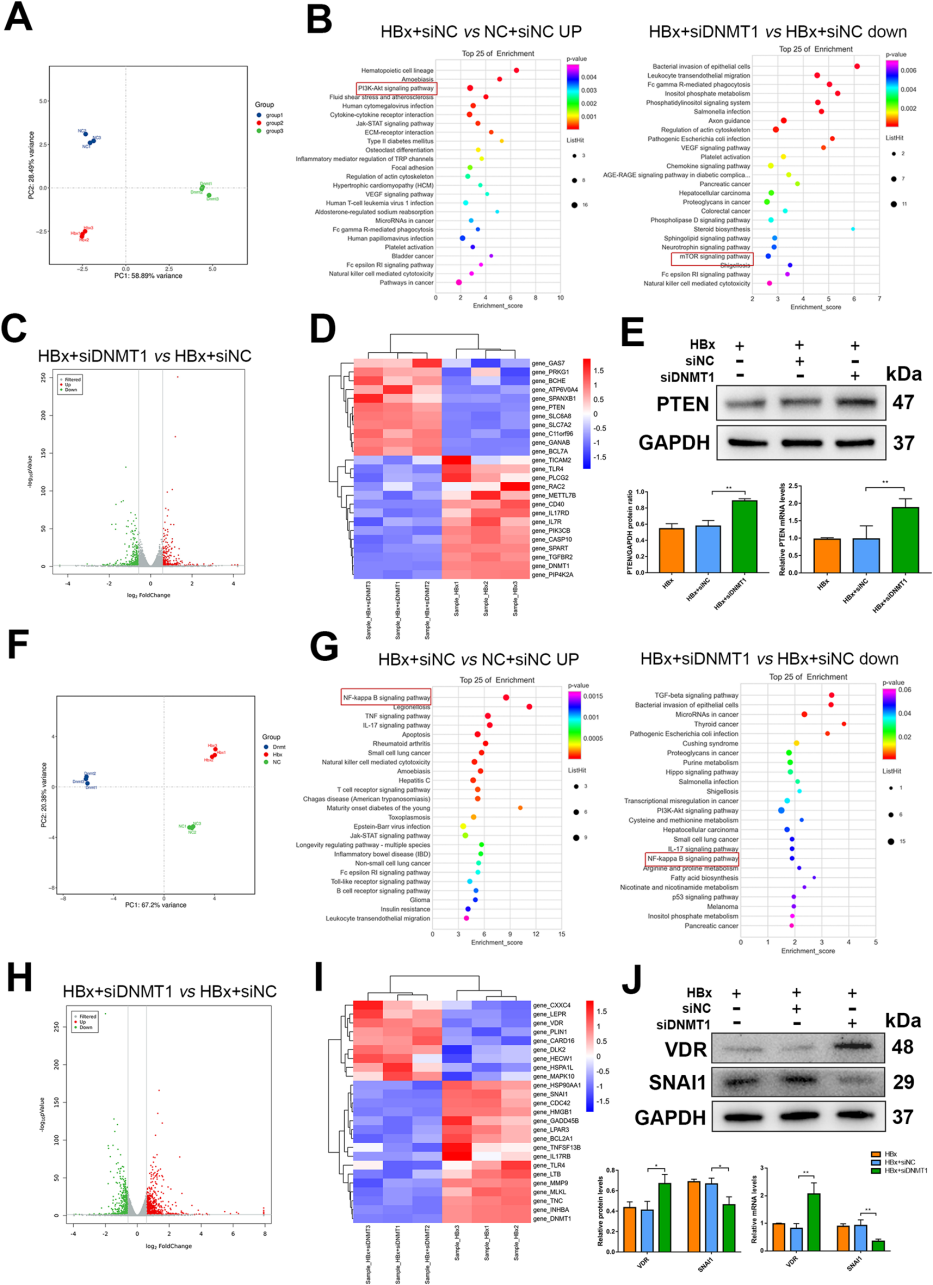
RNA-sequencing after DNMT1 knockdown in HBx-overexpressing renal cells. **A** PCA in HK-2 cells and siNC- or siDNMT1-transfected HBx-HK-2 cells (three replicates). **B** KEGG pathway analyses for identifying potential pathways in HK-2 cells and siNC- or siDNMT1-transfected HBx-HK-2 cells. **C** Differentially expressed genes (79 upregulated and 97 downregulated) identified in the volcano plot for siNC- or siDNMT1-transfected HBx-HK-2 cells. **D** Heatmap showing 25 differentially expressed genes in siNC- or siDNMT1-transfected HBx-HK-2 cells. **E** RT-qPCR and western blotting were performed to confirm the altered mRNA and protein expression of PTEN after knocking down DNMT1. **F** PCA of podocytes and siNC- or siDNMT1-transfected HBx-podocytes with three replicates. **G** KEGG pathway analyses for potential pathways in podocytes and siNC- or siDNMT1-transfected HBx-podocytes. **H** Volcano plot revealed differentially expressed genes (291 upregulated and 131 downregulated) in siNC- or siDNMT1-transfected HBx-podocytes. **I** Heatmap showing 25 differentially expressed genes in siNC- or siDNMT1-transfected podocytes. **J** RT-qPCR and western blotting were performed to confirm the altered mRNA and protein expression of SNAI1 and VDR after knocking down DNMT1. Data are expressed as mean ± SD of 3 separate assays. *P < 0.05; **P < 0.01

Based on the above results, we aimed to detect the genes regulated by DNMT1 in HBx-overexpressing HK-2 cells and human podocytes. We conducted transcriptome sequencing (RNA-seq) in the negative control + siNC (NC + siNC), HBx-overexpressing + siNC (HBx + siNC), and HBx-overexpressing + siDNMT (HBx + siDNMT1) cells. Principal component analysis (PCA) indicated favorable repeatability within groups and significant differences among the three groups in HK-2 cells (Fig. 4A). The Kyoto encyclopedia of genes and genomes (KEGG) enrichment analyses were performed to better comprehend the regulatory mechanism of the potential pathways among the three groups. The PI3K/Akt signaling pathway was significantly upregulated in HBx + siNC group (compared to the NC + siNC group). In contrast, the mTOR signaling pathway, a key pathway downstream of PI3K/Akt signaling, was significantly downregulated in DNMT1 knocked down HBx-HK-2 cells, confirming that DNMT1 mediates its effects through the PI3K/Akt/mTOR pathway in HBx-HK-2 cells (Fig. 4B). The volcano plot showed that 166 differentially expressed genes (DEGs), including 79 upregulated and 97 downregulated genes, were affected by DNMT1 knockdown in HBx-HK-2 cells (Fig. 4C). The heatmap revealed 25 DEGs in siNC- or siDNMT1-transfected HBx-HK-2 cells, thereby demonstrating significant changes in the transcriptome (Fig. 4D). We observed alterations in the expression of PTEN —an important target of the PI3K/Akt/mTOR pathway—at the protein and mRNA levels after DNMT1 knockdown using western blotting and RT-qPCR (Fig. 4E).

PCA of podocytes also showed favorable repeatability within groups and significant differences among the NC + siNC, HBx + siNC, and HBx + siDNMT1 groups (Fig. 4F). KEGG enrichment analyses for potential pathways in podocytes revealed that the NF-κB pathway—a key inflammatory pathway—was significantly upregulated in HBx-podocytes but downregulated after DNMT1 knockdown (Fig. 4G). These results confirmed the anti-inflammatory effects of DNMT1 knockdown. Volcano plot revealed that 422 DEGs, including 291 upregulated and 131 downregulated genes, were affected by DNMT1 knockdown in HBx-podocytes (Fig. 4H). The heatmap showed 25 DEGs in siNC- and siDNMT1-transfected HBx-podocytes (Fig. 4I). The alterations in the protein and mRNA expression of the main inflammation targets and EMT—VDR and SNAI1—were confirmed by western blotting and RT-qPCR after DNMT1 knockdown (Fig. 4J). These results suggest that PTEN and VDR—downstream targets of DNMT1—might play an important role in regulating renal injuries in HBx-overexpressing HK-2 cells and human podocytes, respectively.

### DNMT1 knockdown protects against renal injuries by mediating PI3K/Akt/mTOR and NF-κB signaling

**Fig. 5:**
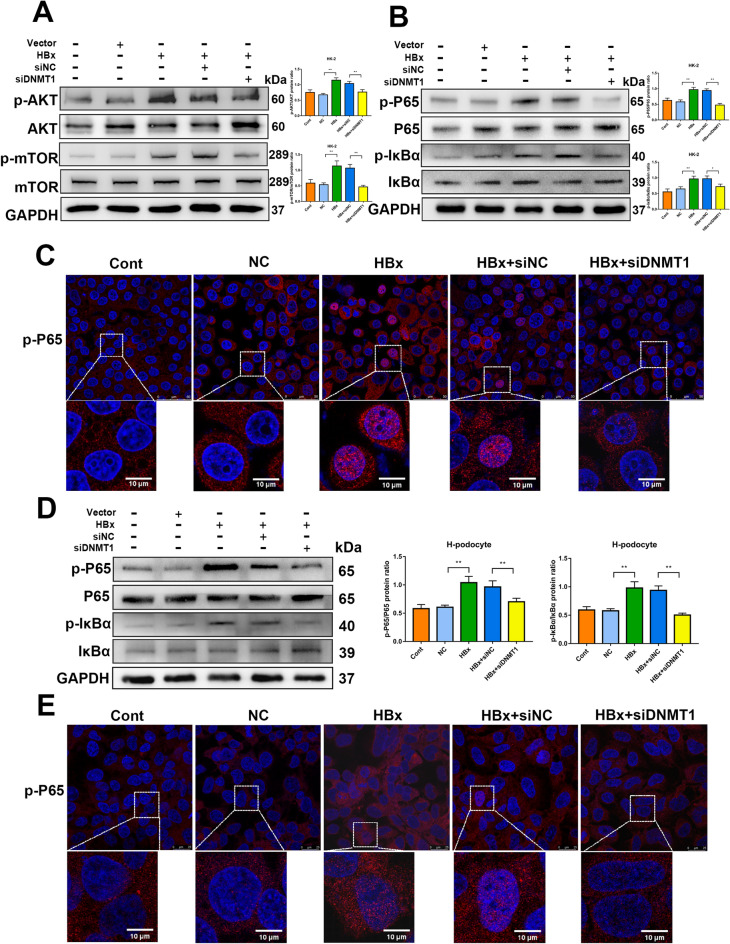
HBx activated PI3K/Akt/mTOR and NF-κB pathways in cultured human podocytes and HK-2 cells. **A** Western blotting evaluating Akt, p-Akt, mTOR, and p-MTOR levels among different groups in HK-2 cells treated with or without inhibition of DNMT1. **B** Western blotting evaluating P65, p-P65, IκBα, and p-IκBα levels in HK-2 cells exposed to siDNMT1 or not across diverse groups. **C** Representative micrographs showing the immunostaining for p-NF-κB P65 within HK-2 cells among different groups. Scale bar: 10 and 50 μm. **D** Western blotting evaluating P65, p-P65, IκBα and p-IκBα levels within podocytes exposed to siDNMT1 or not across diverse groups. **E** Representative micrographs showing the immunostaining for p-NF-κB P65 among different groups in podocytes as indicated. Scale bar: 50 μm and 10 μm. Results are expressed as mean ± SD of 3 separate assays. *P < 0.05; **P < 0.01

Based on the results of RNA-seq, we examined the activation of PI3K/Akt/mTOR and NF-κB signaling in response to HBx overexpression. Western blotting demonstrated that overexpression of HBx distinctly improved the levels of p-AKT and p-mTOR compared to those in the NC group. Nevertheless, DNMT1 inhibition significantly prevented the increase in p-AKT and p-mTOR levels in HBx-HK-2 cells (Fig. 5A). As the NF-κB pathway is a vital inflammatory pathway that functions downstream of the AKT pathway, we examined the effect of DNMT1 on the activation of the NF-κB pathway in HK-2 cells. DNMT1 inhibition reversed the increase in p-P65 and p-IκBα levels in HBx-HK-2 cells (Fig. 5B). To confirm the effect of DNMT1 on the NF-κB pathway in HBx-podocytes, we detected the levels of p-P65, P65, p-IκBα, and IκBα in HBx-podocytes with or without DNMT1 inhibition. Similar results for the NF-κB signaling pathway were observed in HBx-podocytes (Fig. 5D). Immunofluorescence revealed the nuclear translocation of p-P65 in HBx-overexpressing HK-2 cells and human podocytes (Fig. 5C and E). Blocking of DNMT1 significantly attenuated the nuclear translocation of p-P65 (Fig. 5 C and E). Collectively, these data indicated that DNMT1 inhibition attenuated HBx-induced activation of PI3K/Akt/mTOR and NF-κB signaling in vitro.

### Knockdown of DNMT1 reverses PTEN promoter hypermethylation in HBx-HK-2 cells

**Fig. 6:**
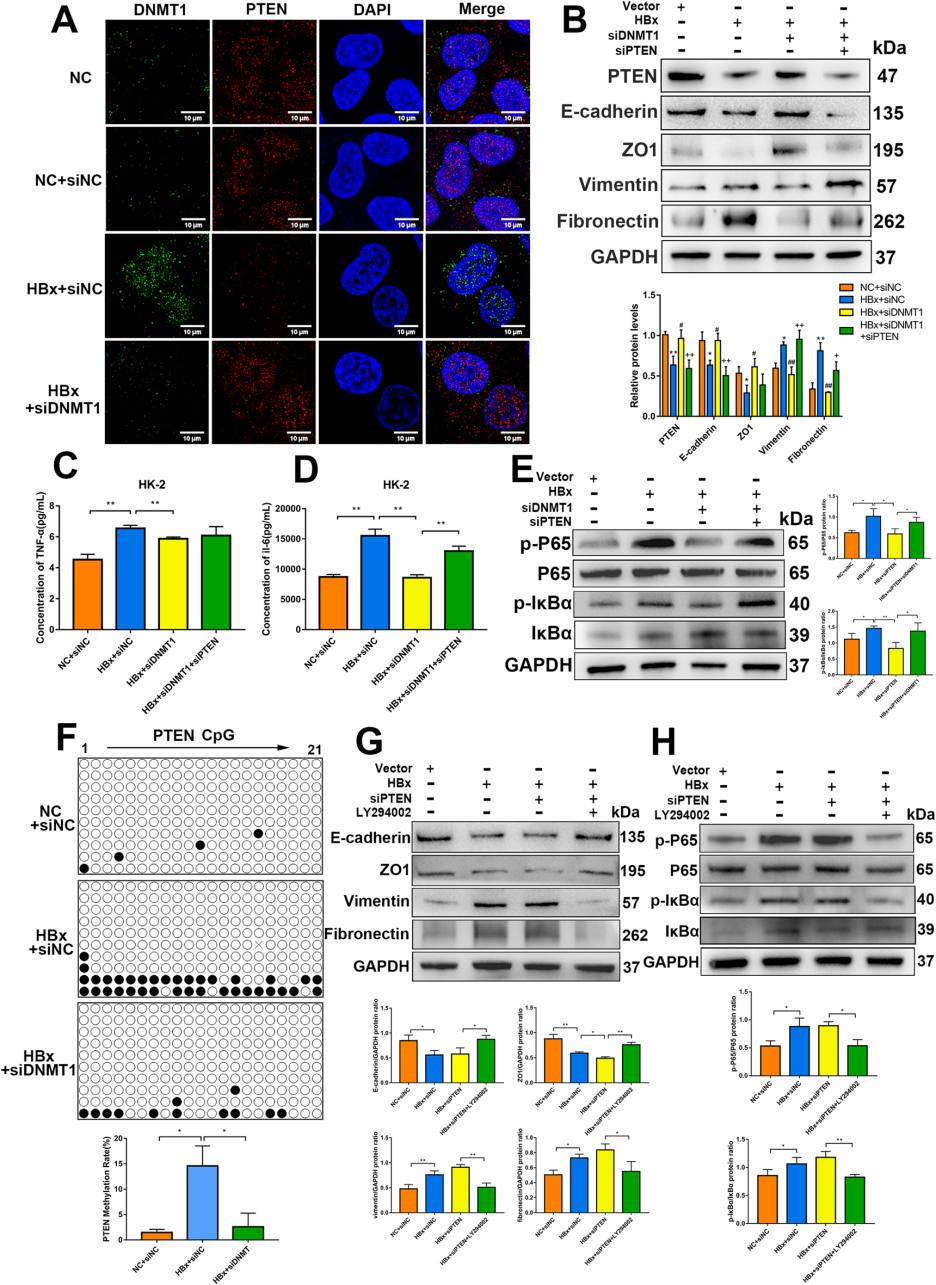
DNMT1 regulated PTEN methylation in kidney injuries in HBx-HK-2 cells. **A** DNMT1 and PTEN levels and localization within HBx-HK-2 cells measured through immunofluorescence. Scale bar: 10 μm. **B** Western blotting for PTEN, E-cadherin, ZO1, Vimentin, and Fibronectin in HBx-HK-2 cells treated with or without inhibition of DNMT1 or PTEN. **C** and **D** Concentrations of IL-6 and TNF-α in HBx-HK-2 cells supernatant exposed to siDNMT1 or siPTEN transfection were detected by ELISA. **E** Western blotting for P65, p-P65, IκBα and p-IκBα in HBx-HK-2 cells treated with or without inhibition of DNMT1 or PTEN. **F** Representative BSP analysis for evaluating the DNA methylation status of the PTEN promoter region (21 CpG dinucleotides) in HK-2 cells and HBx-HK-2 cells transfected with siNC or siDNMT1. Methylated CpGs are indicated as black dots; unmethylated CpGs are indicated as empty dots. **G** Western blotting for E-cadherin, ZO1, Vimentin, and Fibronectin in HBx-HK-2 cells exposed to siPTEN or LY294002. **H** Western blotting for P65, p-P65, IκBα and p-IκBα in HBx-HK-2 cells exposed to siPTEN inhibition or LY294002. Results are expressed as mean ± SD of 3 separate assays. *P < 0.05; **P < 0.01

To obtain additional insights into the possible involvement of DNA methylation alterations in PTEN in HBx-HK-2 cells, we first examined the expression and localization of DNMT1 and PTEN using immunofluorescence. We found that DNMT1 knockdown resulted in the reversal of HBx-induced reduction in PTEN expression. Further, we observed a co-localization between DNMT1 and PTEN (Fig. 6A). To further verify the mechanisms by which DNMT1 affects PTEN expression in HK-2 cells, we successfully silenced PTEN using siRNA-mediated gene silencing. Western blotting revealed that DNMT1 inhibition significantly prevented EMT in HBx-HK-2 cells (Fig. 6B). However, PTEN silencing in the background of DNMT1 knockdown prevented the reversal of EMT (Fig. 6B). ELISA revealed that DNMT1 knockdown reduced TNF-α and IL-6 release, and PTEN silencing partially reversed the reduction in IL-6 release (Fig. 6C and D). PTEN silencing significantly reversed the downregulation of the NF-κB signaling pathway in DNMT1 knocked down HBx-HK-2 cells (Fig. 6E). Then, we checked PTEN promoter methylation in a CpG-rich area using BSP. Methylation analysis of 21 CpG sites revealed that HBx-HK-2 cells displayed increased methylation of the PTEN promoter, whereas DNMT1 knockdown resulted in low methylation levels (Fig. 6F). To further verify the role of PTEN in regulating renal injury, we used the PI3K inhibitor LY294002 to confirm the pathway downstream of PTEN. Western blotting indicated that the PI3K inhibitor LY294002 could markedly enhance the PTEN knockdown‒induced EMT and NF-κB pathway activation (Fig. 6G and H). The above results illustrate that HBx enhances PTEN promoter hypermethylation by upregulating DNMT1, thus upregulating the PI3K/Akt/mTOR and NF-κB pathways in HK-2 cells.

### Knockdown of DNMT1 reverses VDR promoter hypermethylation in HBx-podocytes

**Fig. 7:**
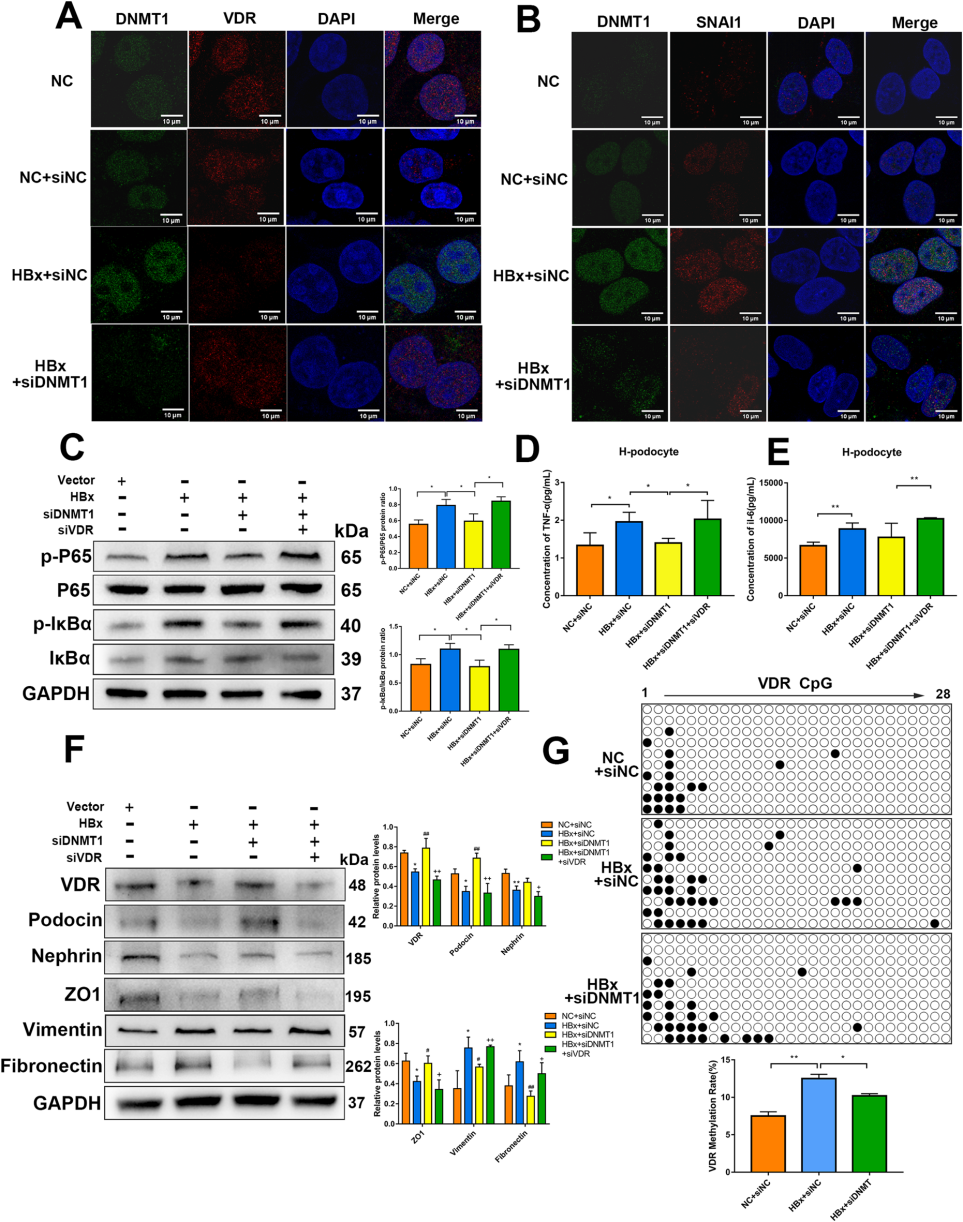
DNMT1 regulated VDR methylation during kidney injuries in HBx-podocytes. **A** The localization and expression of DNMT1 and VDR in HBx-podocytes were determined by immunofluorescence. **B** DNMT1 and SNAI1 levels and localization within HBx-podocytes detected through immunofluorescence. Scale bar: 10 μm. **C** Western blotting for p-P65, P65, p-IκBα, and IκBα in HBx-podocytes treated with or without inhibition of DNMT1 or VDR. **D** and **E** Concentrations of TNF-α and IL-6 in the supernatant of HBx-podocytes with or without transfecting siDNMT1 or siVDR were evaluated by ELISA. **F** Western blotting for VDR, Podocin, Nephrin, ZO1, Vimentin, and Fibronectin in HBx-podocytes treated with or without inhibition of DNMT1 or VDR. **G** Representative BSP analysis for the DNA methylation status of the VDR promoter region (28 CpG dinucleotides) in human podocytes and HBx-podocytes transfected with siNC or siDNMT1. Methylated CpGs are indicated as black dots; unmethylated CpGs are indicated as empty dots. Results are expressed as mean ± SD of 3 separate assays. *P < 0.05; **P < 0.01

DNMT1 downregulated and upregulated VDR and SNAI1, respectively in HBx-podocytes. Immunofluorescence revealed that DNMT1 was upregulated and VDR was downregulated in HBx-podocytes; however, in the background of DNMT1 knockdown, the expression of VDR was remarkably upregulated. Further, DNMT1 and VDR were colocalized in the nucleus (Fig. 7A). DNMT1 knockdown prevented the upregulation of SNAI1 in HBx-podocytes (Fig. 7B). Western blotting and ELISA demonstrated that VDR silencing prevented the reversal of DNMT1 knockdown‒induced NF-κB pathway downregulation and partial proinflammatory cytokine downregulation (Fig. 7C, D, and E). In contrast, DNMT1 knockdown resulted in the downregulation of vimentin and fibronectin and upregulation of nephrin, podocin, and ZO1 in HBx-podocytes, which was reversed upon silencing VDR, consequently contributing to EMT (Fig. 7F). Methylation analysis of 28 CpG sites further revealed that overexpression of HBx significantly increased VDR promoter methylation, and DNMT1 knockdown reversed this increase to the control level (Fig. 7G).

**Fig. 8:**
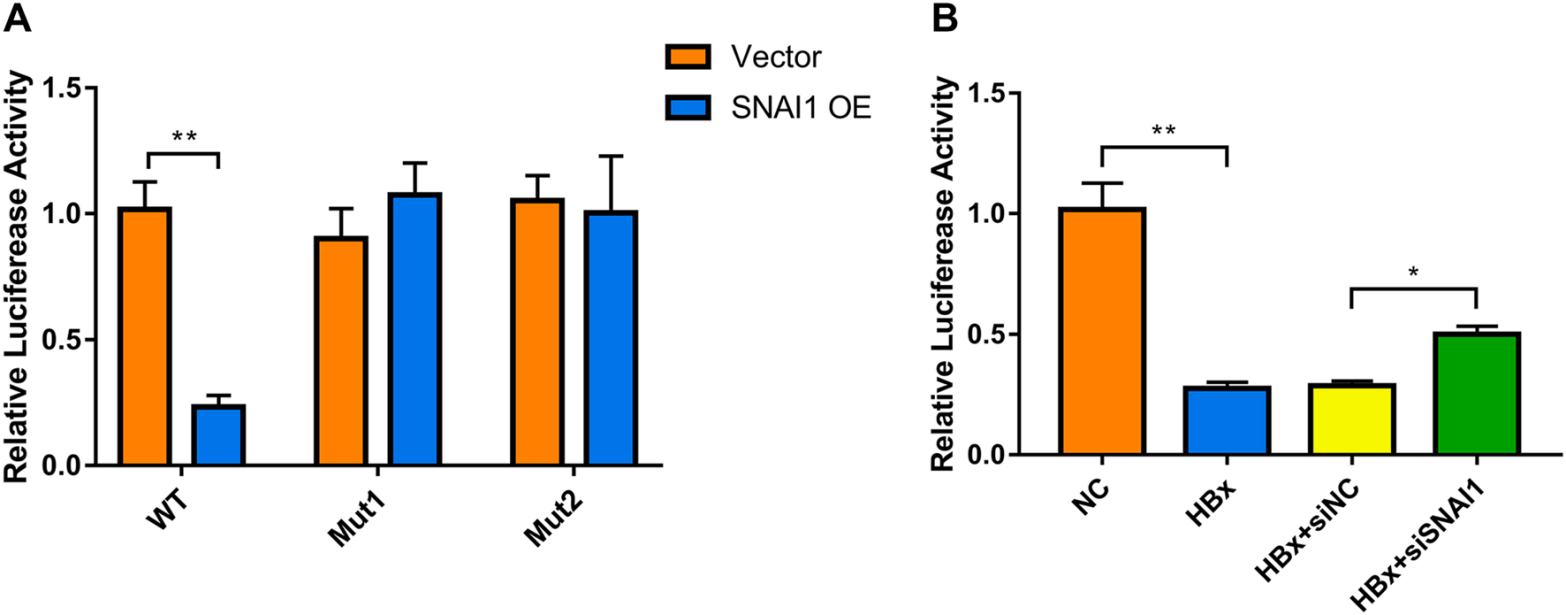
The transcription factor SNAI1 represses VDR in HBx-podocytes. **A** Luciferase activities of podocyte lysates subject to basic vector (Vector) or pcDNA3.1/Flag-Snai1 (SNAI1) transfection with pGL4.10-VDR wild-type promoter (VDR-WT) or VDR mutant promoter (Mut1 and Mut2). Renilla activity was used to normalize firefly activity. **B** HBx-podocytes were transfected with the VDR reporter plasmid and a Renilla luciferase reporter, which were subsequently treated with or without SNAI1 knockdown. Renilla activity was adopted for normalizing firefly activity. Results are expressed as mean ± SD of 3 separate assays. *P < 0.05; **P < 0.01

To explore a direct correlation between SNAI1 function and VDR expression, we evaluated the effect of SNAI1 on the transcriptional activity of the VDR promoter. A dual-luciferase reporter assay was performed to verify the relationship between SNAI1 and VDR. The results showed that SNAI1 decreased the luciferase activity in case of wild-type VDR (WT), but not in case of VDR promoter with a mutation at the predicted SNAI1-binding site (Mut1 and Mut2) (Fig. 8A). Furthermore, luciferase activity associated with the VDR promoter was lowered in HBx-podocytes and increased after SNAI1 knockdown (Fig. 8B). These results suggest that SNAI1 directly modulates the expression of VDR by binding to its promoter, thereby promoting fibrosis in HBx-induced renal injuries.

### DNMT1 inhibitor 5-Aza alleviates kidney injuries in HBV transgenic mice

**Fig. 9:**
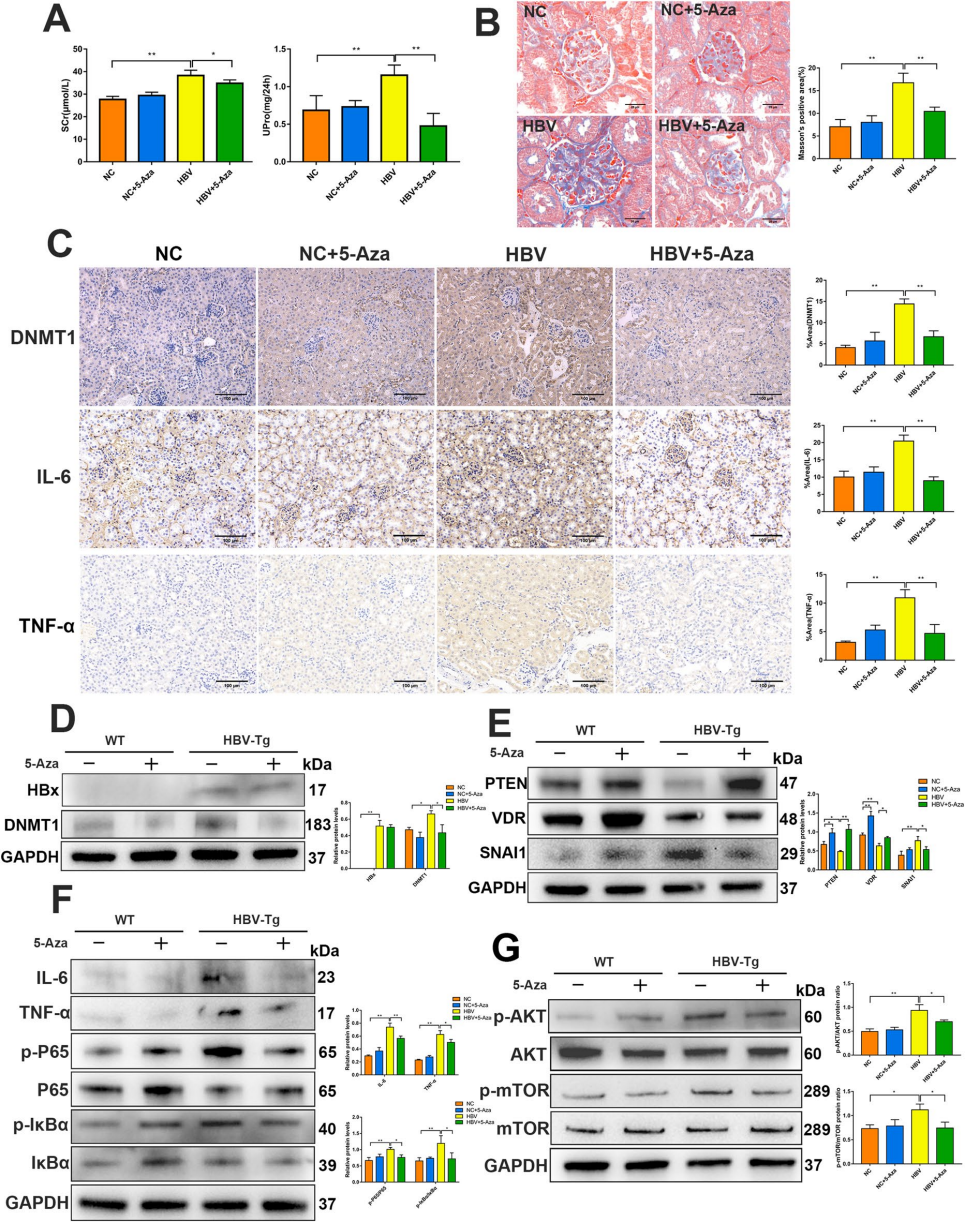
DNMT1 inhibitor 5-Aza protected from renal injuries ***in vivo***. **A** Scr and Upro levels of the C57 + PBS(NC), C57 + 5-Aza (NC + 5-Aza, 1 mg/kg weight), HBV + PBS, HBV + 5-Aza (1 mg/kg weight) groups at 24 weeks were measured. **B** Representative Masson’s stained kidney sections of different groups. Scale bar: 25 μm. **C** Immunohistochemistry for DNMT1, IL-6, and TNF-α in the renal cortex from experimental mice. Scale bar: 100 μm. **D** Western blotting to evaluate HBx and DNMT1 expression in the kidneys from the experimental mice. **E** Western blotting to evaluate PTEN, VDR, and SNAI1 expression in the kidneys from the experimental mice. **F** Western blotting on IL-6, TNF-α, P65, p-P65, IκBα and p-IκBα levels in the kidneys from the experimental mice. **G** Western blotting to evaluate Akt, p-Akt, mTOR, and p-mTOR levels in the experimental mouse kidneys. Results are expressed as mean ± SD of 3 separate assays. *P < 0.05; **P < 0.01

To investigate whether DNMT1 could regulate renal injury in vivo, we used HBV transgenic (HBV-Tg) mice and wild-type (NC) C57BL/6 mice with or without gavage with the DNMT1 inhibitor 5-Aza. Scr and 24 h Upro levels were also significantly reduced upon 5-Aza treatment (Fig. 9A). Histological examination of the kidney by Masson’s trichrome staining revealed that 5-Aza decreased mesangial matrix expansion and fibrosis in the glomeruli of HBV-Tg mice (Fig. 9B). IHC of the renal cortex revealed that the expression of DNMT1, TNF-α, and IL-6 was upregulated in HBV-Tg mice, and this upregulation was inhibited by 5-Aza treatment (Fig. 9C). Western blotting revealed that the expression of HBx and DNMT1 was elevated in the kidneys of HBV-Tg mice. However, 5-Aza markedly decreased DNMT1 expression but did not reduce HBx expression (Fig. 9D). These results suggested that the DNMT1 inhibitor might attenuate renal injuries independently of the direct downregulation of HBx, and instead might regulate the specific downstream pathway in the kidneys of HBV-Tg mice. To verify the downstream targets of DNMT1 identified in vivo, we examined the expression of PTEN, VDR, and SNAI1 in the kidneys of different groups. Western blotting revealed that the 5-Aza markedly reversed the upregulation of SNAI1 and downregulation of PTEN and VDR in the kidneys of HBV-Tg mice (Fig. 9E). Mechanistically, 5-Aza inhibited the release of inflammatory mediators and the activation of the NF-κB and Akt/mTOR pathways (Fig. 9F and G). Altogether, these data further demonstrate that the DNMT1 inhibitor‒mediated restoration of PTEN and VDR levels is a critical mechanism that could serve as a potential therapeutic strategy for renal protection in HBV-Tg mice.

## Discussion

It has been reported that HBx mediated the establishment and maintenance of the chronic carrier state. Currently, the mechanisms regulating HBx-induced inflammatory injury and fibrosis are mostly concentrated in the liver [21]. The role of HBx in renal inflammation and fibrosis during HBV-GN is not well documented. In addition to bone marrow-derived fibroblasts, resident fibroblasts generated during EMT have been demonstrated to be an important mechanism responsible for kidney fibrosis progression [22,23]. HBV infection can directly cause inflammatory cell infiltration and immune disorders, eventually leading to kidney fibrosis [3, 9,13]. Thus, we reasoned that HBx could enhance the production of inflammatory cytokines, such as TNF-α and IL-6, and decrease that of podocyte slit diaphragm proteins and renal tubular epithelial proteins. In this study, analysis of renal biopsy and HBV-Tg mouse renal tissue samples revealed that HBx expression was positively associated with the release of proinflammatory mediators, whereas it was inversely associated with expression of renal tubular epithelial and podocytes marker proteins, demonstrating direct virus-induced pathological alterations in the kidney.

HBx, a pleiotropic protein involved in multiple pathogenic processes, can initiate many epigenetic modifications, including DNA methylation, histone modifications, and chromatin remodeling [24,25]. Aberrant DNA methylation patterns have been associated with the progression of various kidney diseases [26,27]. In this study, we discovered that DNMT1 expression was upregulated in the kidneys of HBV-GN patients with accompanying renal injuries. Previous study has uncovered the role of DNMT1 and DNMT3a upregulation in enhancing the methylation of the Klotho promoter in mice exposed to adenine treatment [28]. However, our results showed that DNMT1 was the main subtype to promote the secretion of inflammatory mediators and EMT process without altering the levels of DNMT3a and 3b in HBx-induced renal injury. Consistent with our study, DNMT1 has been reported as a unique DNMT subtype that mediates hypermethylation of the RasalI promoter, thereby leading to persistent renal fibrosis [29]. HBx can activate DNMT1 expression through the E2F1 pathway [30], further supporting our hypothesis. It is currently unknown whether the discrepancies were caused by the different disease models, which warrants further investigation.

The molecular events involved in the epigenetic regulation of HBx-induced kidney injury are not fully understood. DNA methylation of promoters can reprogram gene expression by silencing gene expression, thereby regulating ongoing kidney damage. Furthermore, DNMT1 knockdown did not affect HBx expression in vivo or in vitro, demonstrating that its renoprotective effect was mainly dependent on downstream signaling pathway rather than directly affecting HBV. PTEN is a negative regulator of the Akt and mTOR pathways and its expression is controlled by several pathologic processes and cellular factors including epigenetic mechanisms [31,32]. PTEN downregulation may contribute to immune dysregulation, leading to glomerular hypertrophy, tubular hypertrophy, fibrotic reprogramming, and epithelial dedifferentiation through Akt activation[33,34]. Genetic suppression of PTEN could inhibit its EMT-promoting role, causing alleviation of kidney damage, thereby improving the survival rates [35]. Previous research found that PTEN protein was decreased when epigenetic expression of multiple miRNAs and rescued by knocking down relative miRNA in HBx induced pathological state [36,37]. HBx interrupted the balance between miRNA and PTEN, which contributed to the development of multiple diseases [38,39]. These data suggest that HBx uses more than one epigenetic mechanism to induce cell injuries. In this study, we presented here a novel regulation mechanism between DNMT1 and PTEN in HBx induced HK-2 cells. We confirmed that HBx induced PTEN promoter methylation to promote inflammation and fibrogenesis in cultured HK-2 cells, a phenomenon that was partially reversed in the background of DNMT1 knockdown.

Furthermore, we examined the effect of DNMT1 on the downstream pathway of PTEN. At the molecular level, silencing of PTEN could reverse the regulation pattern of DNMT1 in HBx-HK-2 cells by upregulating the PI3K/Akt/mTOR and NF-κB pathways. Similar to our findings, Xu et al. confirmed that the m6A modification-related PI3K/Akt pathway modulated by methylase METTL14 through PTEN had an effect on HDAC5-induced EMT in renal tubular cells exposed to high glucose (HG) treatment [40]. DNMT1 could downregulate PTEN, while PTEN overexpression reduced inflammation and activated fibroblast-like synoviocytes via AKT signaling in rheumatoid arthritis [41]. Moreover, our results indicated that the PI3K/Akt inhibitor LY294002 attenuated PTEN downregulation-induced EMT and NF-κB activation, thereby demonstrating that activation of the PI3K/Akt pathway in response to PTEN loss could result in the activation of the downstream proinflammatory NF-κB pathway. NF-κB is a prototypical proinflammatory transcription factor that binds to the IκB protein in the cytoplasm. IκB phosphorylation and ubiquitination result in its degradation and this is followed by p65 phosphorylation, translocation to the nucleus, and subsequent transcription of several target genes [42]. The PI3K/Akt/NF-κB pathway also mediates EMT in many cancer cells [43–46]. Increasing evidence reveal that Akt/mTOR and NF-κB activation upregulates renal inflammation and dramatically promotes renal interstitial fibrosis and fibroblast activation [47–50]. And upregulation of autophagy upon PI3K/Akt/mTOR pathway inhibition can significantly stabilize IκB-α and reduce the expression of TNF-α-induced EMT [51].

The other downstream target, VDR, were studied to explore the underlying mechanism of increased Dnmt1 in HBx-podocytes. VDR, a nuclear receptor superfamily member, harbors a ligand-independent cell-specific transcriptional activation autonomic regulatory domains. In addition to classical regulation of the calcium and phosphorus balance, relevant results have shown that VDR plays an important role in cell differentiation, immunological regulation, and inflammation. Clinical research indicated that genetic variants of the VDR genes were associated with increased susceptibility to HBV-related hepatocellular carcinoma [52]. HBV downregulates VDR expression, thereby avoiding the immunological defense system [53]. Evidence from relevant renal studies has revealed that VDR agonists could be used to reduce proteinuria via downregulating renin transcription. Nonetheless, VDR agonists do not upregulate VDR expression, and adding a DNA methylation inhibitor to VDR agonists amplifies VDR levels. The epigenetic status of the VDR-targeted gene transcription start sites was regulated by altering the DNA methylation and H3K4me2/H3K9Ac levels within prostatic cancer [54]. Consistently, our results verified that HBx increased VDR promoter hypermethylation and silencing of VDR could reverse the downregulation of EMT and inflammatory mediator production after DNMT1 knockdown.

To further identify the precise signaling pathway by which DNMT1 aids HBx-induced podocytes injuries. Genetic evidence indicates that master regulators of EMT, such as TWIST and SNAI1, play important roles in kidney fibrosis progression. Our luciferase reporter assay showed that VDR was a direct SNAI1 target, and silencing of SNAI1 partially enhanced the VDR activity in HBx-podocytes. Chandel et al. proved that human immunodeficiency virus induced SNAI1 repressor complex formation, consisting of DNMT1-regulated VDR expression in podocytes, accounted for the increased CpG methylation at the VDR promoter and epigenetic alterations associated with the alleviation of podocyte injuries [55]. However, the specific SNAI1 mechanism in VDR requires further investigation. VDR agonists can inhibit NF-κB activity and reduce renal podocyte inflammation in some experimental renal disease models (e.g., azithromycin-induced renal injury), thereby alleviating renal injury [56]. Increased levels of Sp1 and NF-κB-p65 bound to the DNMT1 promoter region in podocytes under diabetic conditions, and this resulted in hypermethylation of podocyte slit diaphragm proteins which exacerbated podocyte damage [57]. Thus, we hypothesized that the NF-κB pathway as a key regulator in DNMT1-mediated regulation of podocyte injuries was further confirmed by concurrently knocking down VDR and DNMT1.

Consistent with the results of in vitro experiments, our research also revealed that pretreatment with the DNMT1 inhibitor 5-Aza mitigated renal injuries, improved kidney function and downregulated PI3K/Akt/mTOR and NF-κB pathways in the kidneys of HBV-Tg mice, and this renoprotective effect was independent of elimination of virus. Although our current study has conducted an in-depth exploration of the role of DNMT1 in HBV-GN, some limitations of this study need to be addressed. First, HBx overexpression of renal cells could not completely simulate all pathological processes of HBV damage to renal cells. Second, DNMT inhibitor was used to verify the systemic effects of vital targets in vivo experiments, but there was no specific knockout or knockin model for key molecules such as HBx and DNMT1 in kidney tissue. Third, further clinical validation of developed in vivo HBV-Tg model and clinical efficacy of DNMT inhibitor in HBV-GN patients.

## Conclusions

**Fig. 10:**
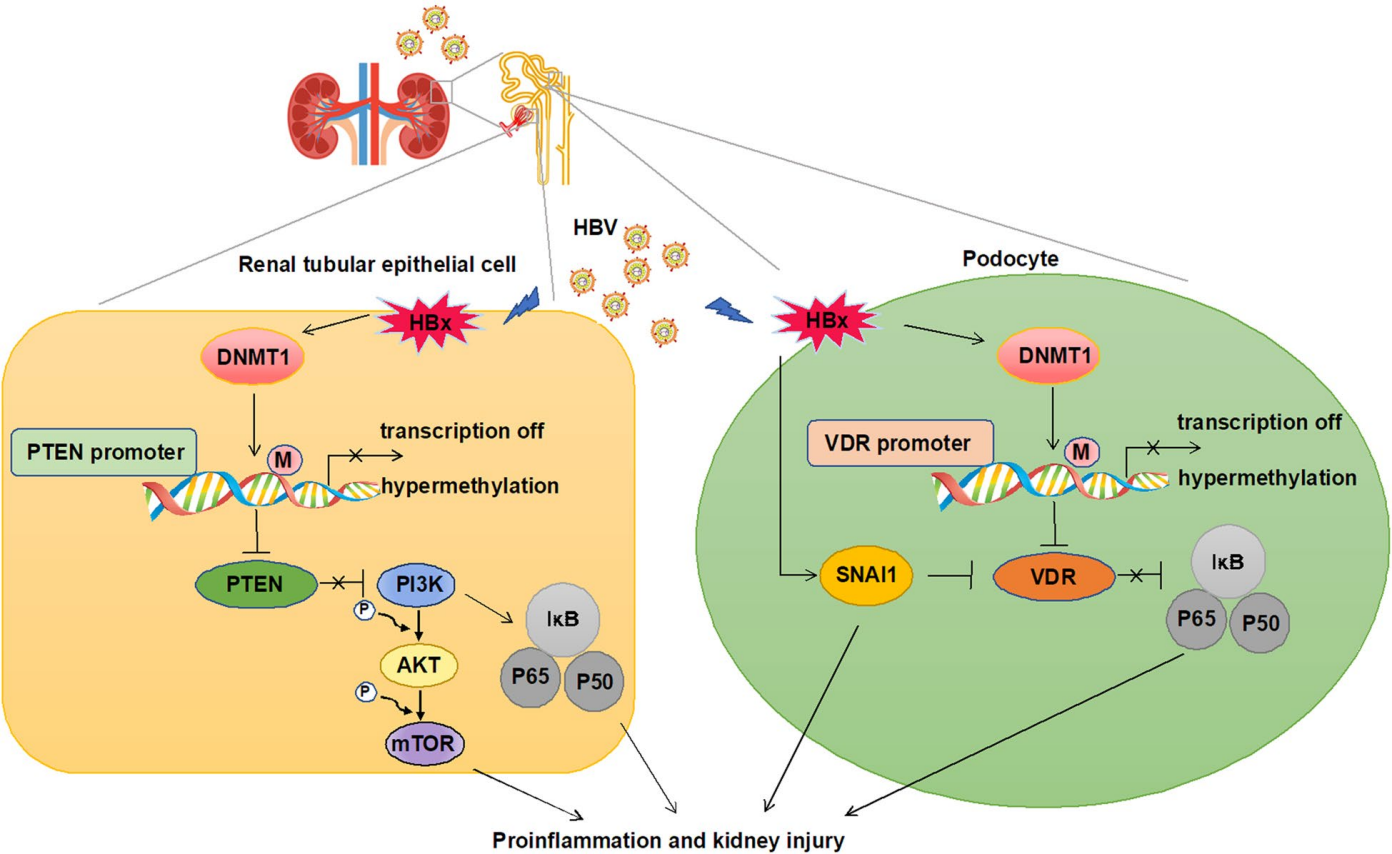
The proposed mechanism of DNMT1 silencing related to protecting against HBx-induced kidney injuries. DNMT1 knockdown protects against renal injuries mediated by PI3K/Akt signaling and VDR signaling by regulating the DNA methylation status of PTEN and VDR in HBV-GN

In conclusion, we have demonstrated an epigenetic mechanism by which HBx induces renal EMT and inflammation and uncovered a vital characteristic of the DNMT1 inhibitor with respect to preventing and treating HBV-GN. Our findings provide strong evidence that DNMT1 is upregulated in HBx-overexpressing HK-2 cells and podocytes, leading to PTEN and VDR promoter hypermethylation, respectively, and subsequent activation of the PI3K/Akt/mTOR and NF-κB signaling pathways. Thus, DNMT1 may be exploited as a novel therapeutic target for intervention in HBV-GN progression (Fig. 10).

## Supplementary Information

**Additional file 1: Table S1.** The siRNA sequences that target HBx, PTEN, VDR and GAPDH are listed below. **Table S2.** Primer sequences used for quantitative Real-time PCR are listed below. **Table S3**. Primer sequences used for bisulfite sequencing PCR are listed below. **Figure S1.** DNMT1, PTEN and VDR knockdown in HK-2 cells and human podocytes. (A) HK-2 cells and human podocytes were treated with Lipo3000 (NC) or transfected with control siRNA (siNC) as negative, GAPDH siRNA (siGAPDH) as positive control or DNMT1 siRNAs (siDNMT1) for 48 h. DNMT1 protein levels were determined by Western blotting. (B) HK-2 cells were transfected with control siRNA (siNC) as negative or PTEN siRNAs (siPTEN) for 48h. PTEN protein levels were determined by Western blotting. (C) Human podocytes were transfected with control siRNA (siNC) as negative or VDR siRNAs (siVDR) for 48 h. VDR protein levels were determined by Western blotting. Data were represented as mean ± SD from three independent experiments. **P < 0.01 vs siNC group.

## Data Availability

The data that support the findings of this study are not publicly available due to technical or time limitations but are available from the corresponding author on reasonable request.

## Abbreviations

BSP: Bisulfite-sequencing PCR
DEGs: Differentially expressed genes
DNMT1: DNA methyltransferase 1
EMT: Epithelial-mesenchymal transition
GO: Gene ontology
HBsAg: Hepatitis B surface antigen
HBV-GN: Hepatitis B virus-associated glomerulonephritis
HBx: Hepatitis B virus X protein
KEGG: Kyoto Encyclopedia of Genes and Genomes
NF-κB: Nuclear factor-kappa B
PTEN: Phosphatase and tensin homolog
SNAI1: Snail family transcriptional repressor 1
Scr: Serum creatinine
TRAIL: Tumor necrosis factor‒related apoptosis-inducing ligand
Upro: Urinary protein
VDR: Vitamin D receptor
5-Aza: 5-azacytidine
