# Supplementary material for: DNA methyltransferase 1 knockdown reverses PTEN and VDR by mediating demethylation of promoter and protects against renal injuries in hepatitis B virus-associated glomerulonephritis
Source: Cell Biosci. 2022 Jun 28;12:98. doi: 10.1186/s13578-022-00835-1 (PMC9238139; doi:10.1186/s13578-022-00835-1)
Supplement: Supplementary file 1 — Additional file 1: Table S1. The siRNA sequences that target HBx, PTEN, VDR and GAPDH are listed below. Table S2. Primer sequences used for quantitative Real-time PCR are listed below. Table S3. Primer sequences used for bisulfite sequencing PCR are listed below. Figure S1. DNMT1, PTEN and VDR knockdown in HK-2 cells and human podocytes. (A) HK-2 cells and human podocytes were treated with Lipo3000 (NC) or transfected with control siRNA (siNC) as negative, GAPDH siRNA (siGAPDH) as positive control or DNMT1 siRNAs (siDNMT1) for 48 h. DNMT1 protein levels were determined by Western blotting. (B) HK-2 cells were transfected with control siRNA (siNC) as negative or PTEN siRNAs (siPTEN) for 48h. PTEN protein levels were determined by Western blotting. (C) Human podocytes were transfected with control siRNA (siNC) as negative or VDR siRNAs (siVDR) for 48 h. VDR protein levels were determined by Western blotting. Data were represented as mean ± SD from three independent experiments. **P < 0.01 vs siNC group. [file 13578_2022_835_MOESM1_ESM.docx]

**Table S1.** The siRNA sequences that target HBx, PTEN, VDR and GAPDH are listed below. siSNAI1 was purchased from Santa Cruz (sc-38398, CA, USA)

| Gene | siRNA sequence |
| --- | --- |
| Human siDNMT1 | 5'-GGAUGAGUCCAUCAAGGAATT-3' |
|  | 5'-UUCCUUGAUGGACUCAUCCTT-3' |
| Human siVDR | 5'-GUGCCAUUGAGGUCAUCAUTT-3' |
|  | 5'-AUGAUGACCUCAAUGGCACTT-3' |
| Human siPTEN | 5'-GGCUAAGUGAAGAUGACAATT-3' |
|  | 5'-UUGUCAUCUUCACUUAGCCTT-3' |

**Table S2.** Primer sequences used for quantitative Real-time PCR are listed below.

| Genes | Sequences |
| --- | --- |
| HBx | Primer F 5'-GGGTCGCTTGGGACTCTCTC-3' |
|  | Primer R 5'-CGGCAGATGAGAAGGCACAG-3' |
| Human PTEN | Primer F 5'-GACCAGAGACAAAAAGGGAGTA-3' |
|  | Primer R 5'-ACAAACTGAGGATTGCAAGTTC-3' |
| Human VDR | Primer F 5'-AAAGGTCATTGGCTTTGCTAAG-3' |
|  | Primer R 5'-CTTGACTTCAGCAGTACGATCT-3' |
| Human SNAI1 | Primer F 5'-CCTCGCTGCCAATGCTCATCTG-3' |
|  | Primer R 5'-AGCCTTTCCCACTGTCCTCATCTG-3' |
| Human GAPDH | Primer F 5'-GTCTCCTCTGACTTCAACAGCG-3' |
|  | Primer R 5'-ACCACCCTGTTGCTGTAGCCAA-3' |

**Table S3.** Primer sequences used for bisulfite sequencing PCR are listed below.

| Gene | Primers | Product size (bp) |
| --- | --- | --- |
| PTEN | 5'-GAGTAAGGAGTGAGTTTTAGGTTTTA-3' | 351 |
|  | 5'-TAAAAAACTTTCCAAATTCCC-3' |  |
| VDR | 5'-GAGTTYGGGATTTTTTATTYGT-3' | 399 |
|  | 5'-CCACAAATCCAATCCTCTCTT-3' |  |

**
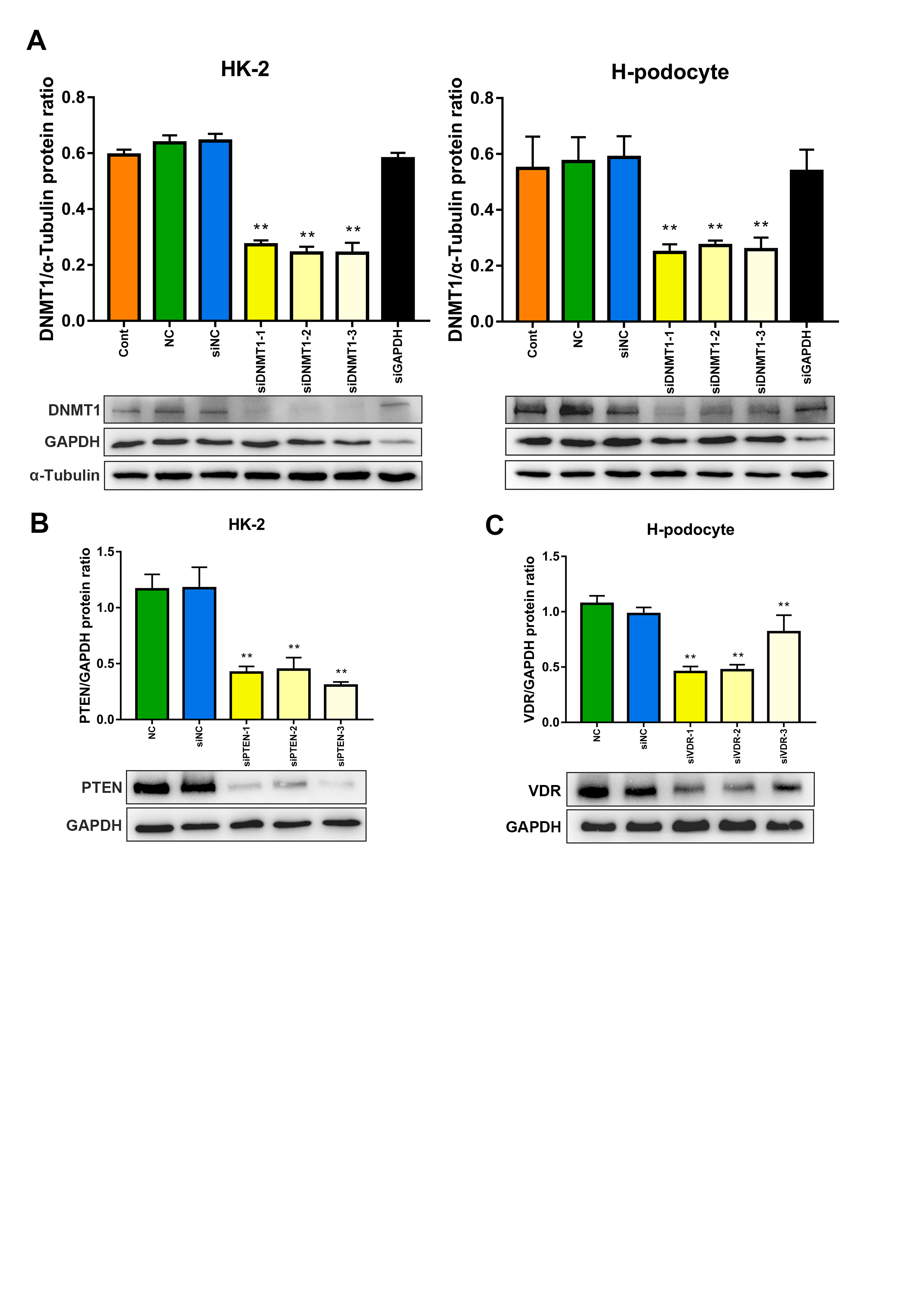
**

**Figure.S1** **DNMT1, PTEN and VDR knockdown in HK-2 cells and human podocytes.** (A) HK-2 cells and human podocytes were treated with Lipo3000 (NC) or transfected with control siRNA (siNC) as negative, GAPDH siRNNA (siGAPDH) as positive control or DNMT1 siRNAs (siDNMT1) for 48 h. DNMT1 protein levels were determined by Western blotting. (B) HK-2 cells were transfected with control siRNA (siNC) as negative or PTEN siRNAs (siPTEN) for 48h. PTEN protein levels were determined by Western blotting. (C) Human podocytes were transfected with control siRNA (siNC) as negative or VDR siRNAs (siVDR) for 48h. VDR protein levels were determined by Western blotting. Data were represented as mean ± SD from three independent experiments. ^**^*P* < 0.01 *vs* siNC group.
